# Correlation between the methylation of SULF2 and WRN promoter and the irinotecan chemosensitivity in gastric cancer

**DOI:** 10.1186/1471-230X-13-173

**Published:** 2013-12-23

**Authors:** Lin Wang, Li Xie, Jun Wang, Jie Shen, Baorui Liu

**Affiliations:** 1 Jiang Su Province Geriatric Institute, Jiang Su Province Geriatric hospital, Nanjing, China; 2 The Comprehensive Cancer Centre of Drum Tower Hospital, Medical School of Nanjing University, Clinical Cancer Institute of Nanjing University, Nanjing, China

**Keywords:** Gastric cancer, SULF2, WRN, Methylation, Irinotecan

## Abstract

**Background:**

At present, no study has compared the correlation between SULF2, WRN promoter methylation and clinicopathological parameters of patients with gastric cancer and the sensitivity to irinotecan (CPT-11).

**Methods:**

We collected 102 fresh tumor tissues from pathologically diagnosed gastric carcinoma patients. Methylation specific PCR was used to detect the promoter methylation of SULF2 and WRN. The chemosensitivity of irinotecan to gastric tomor was tested by MTT. Then we compared the chemosensitivity difference of the methylated group with unmethylated group.

**Results:**

The rates of SULF2, WRN methylation were 28.3% (29/102) and 23.6% (24/102), separately. Patients with SULF2 methylation were more sensitive to CPT-11 than those without SULF2 methylation (*P* < 0.01). Patients with both SULF2 and WRN methylation were also more sensitive to CPT-11 than others ( *P* < 0.05).

**Conclusion:**

SULF2 and WRN promoter methylation detection indicates potential predictive biomarkers to identify and target the most sensitive gastric cancer subpopulation for personalized CPT-11 therapy.

## Background

Gastric cancer is a highly aggressive disease for most population and usually diagnosed at an advanced stage. It is the fourth most common cancer and second leading cause of death from cancer throughout the world. The incidence varies up to 10-fold across the world with the greatest percentage in China, followed by South Korea, South American countries and Japan [1]. Irinotecan (CPT-11, Campto) is an S-phase-specific, semisynthetic derivative of camptothecin which interferes with DNA replication and cell division through its potent interaction with the enzyme topoisomerase I. CPT-11 is frequently used in the treatment of gastric cancer, and showes a good response rate varying from 14% to 23% as single agent and 45% to 70% in combination, with a median time to progression of 3 months in single agent and 4–6 months in combination [2].

Recent studies suggested that the methylation of the heparan sulfate 6-O-endosulfatase (SULF2) promoter is associated with better survival of lung adenocarcinoma patients, and silencing SULF2 through methylation could increase sensitivity to topoisomerase-1 inhibitors such as camptothecin (CPT) chemotherapy [3].

WRN gene is a RecQ family member with both exonuclease and helicase activities. WRN CpG island hypermethylation is a common event in epithelial and mesenchymal tumorigenesis. Besides, WRN hypermethylation in colorectal tumors is a predictor of good clinical response to irinotecan, which is commonly used in the treatment in colorectal cancer [4]. However, up to now, the correlation between SULF2, WRN promoter methylation and the chemosensitivity of irinotecan in gastric cancer has not been studied yet.

In the present work, we collected 102 fresh tissues from pathologically diagnosed gastric carcinoma patients to evaluate the potential correlation. We used methylation specific PCR to detect the promoter methylation of SULF2 and WRN, and adopted the HDRA method to test the chemosensitivity of irinotecan. Also the association of patients’ clinicopathological parameters and methylation status of SULF2 and WRN was analyzed.

## Methods

### Patients and samples preparation

All specimens and relevant clinical data were obtained from the Department of Oncology and General Surgery of Drum Tower Hospital, from 2009 through 2011. There were 75 men and 27 women with a median age of 62 years (range: 29–83 years). Demographic variables including age, gender and stage of gastric cancer are completed. Tumors were staged according to the criteria of the 2003/AJCC staging system for gastric cancer. Each tumor was considered suitable for the study based on the presence of 80% tumor cells. The patients did not undergo chemotherapy or radiotherapy prior to surgery. Informed consent was obtained from all patients and the protocols for our study were approved by the Human Research Protective Committee of Drum Tower Hospital.

### Gastric cancer histoculture drug response assay (HDRA)

Cancerous portions of the specimens were scissor-minced into pieces of approximately 10 mg, placed on a collagen gel-coated well in a 24-well plate, and incubated for 7 days at 37°C in the presence of irinotecan (CPT-11, HengRui, China). Four replicate cultures were evaluated for irinotecan of 20 μg/mL [5]. 7 days later, it was added with 3-(4, 5-Dimethyl-2-thiazotyl)-2, 5-diphenyl-2H-tetrazoliumb-romide (MTT) (5 mg/mL)(Sigma, USA) and 100 μL type I collagenase(1 mg/mL)(Sigma, USA),incubated for another 24 hours. After removal of the medium, formazan was extracted with 0.5 mL of 100% dimethyl sulfoxide (Lingfeng, China) and the absorbance of the solution in each well was read at 490 nm with a microplate reader. The absorbance of tumor tissue was calculated from the mean absorbance of tissue from four culture wells and tumor-tissue weight determined prior to culture.

Inhibition rate (I.R.) (%) = (1-mean absorbance per gram of treated tumor/mean absorbance per gram of control tumor) × 100. In the treatment of gastric cancer, the clinical response rate of irinotecan is lower than 30% as single agent [2]. So in our study, the cut-off value of inrinotecan sensitivity was set at 30% of all samples. Therefore, 30 samples (30.00% of all) were defined as sensitive to irinotecan, and 72 samples (70.00% of all) were defined as resistent, compared to untreated controls.

### DNA extraction and modification

Other tissues were fixed in formalin, dehydrated in alcohol and xylene, and embedded in paraffin. Briefly, sections from paraffin-embedded tissue blocks were obtained. After isolation of DNA, we performed bisulfite treatment. The DNA was then chemically modified by sodium bisulphite to convert all unmethylated cytosines to uracils while leaving methylcytosines unaltered. Then they were stored at −20°C.

### Methylation-Specific Polymerase Chain Reaction (MSP)

The fluorescence-based real-time PCR assay was used in the detection of methylated DNA.

SULF 2: Each 20 μL PCR reaction contained modified DNA 2 uL, SYBR GREEN PCR Mix (TaKaRa, Japan) 10 uL, water 7.7 uL, and primers 0. 15 μL (10 μmol/ L). The mixture was heated for 10 min at 95°, followed by 45 amplification cycles: annealing (59°C for 30 s), extension (72°C for 30 s) and denaturation (95°C for 30 s). Primers for methylated PCR: SULF2 F:5′ TAAGTGTTTTTTTTATAGCGGC 3′, SULF2 R:5′TACCGTAATTTCCGCTATC 3′, Primers for unmethylated PCR: SULF2 F 5′ GTTTATAAGTGTTTTTTTATAGTGGT 3′, SULF2 R: 5′TACCATAATTTCCACTATCCCT 3′.

WRN: Each 20 μL PCR reaction contained modified DNA 2 uL, SYBR GREEN PCR Mix 10uL, water 7.7 uL, and primers 0. 15 μL ( 10 μmol/L). The mixture was heated for 10 min at 95°C, followed by 45 amplification cycles: annealing (56°C for 30 s), extension (72°C for 30 s) and denaturation (95°C for 30 s). Primers for methylated PCR: WRN F: 5′ CGGGTAGGGGTATCGTTCGC 3′,WRN R:5′CGATATCCGAAATCAAACGACG 3′, Primers for unmethylated PCR: WRN R 5′ GTAGTTGGGTAGTAGGGGTATTGTTTGT 3′, WRN F: 5′CCAATATCCAAAATCAAACAACAAC 3′.

SssI-treated human genomic DNA (presumably fully methylated) and reagent blanks were used as positive and negative controls in each experiment. All tests were performed in duplicate.

### Data analysis

Results were analyzed using SPSS 19.0. Patients’ clinicopathologic characteristics, including age, gender, histology type, lymphatic invasion, grade and TNM Stage are summarized with mean and standard deviation for continuous variables and proportions for categorical variables. *χ*2 test was used to assess the association between methylation and patient clinicopathologic characteristics. However, there was no significant association between clinical characteristics and WRN, SULF2 methylation (Table 1). And *t* test was used to compare the relationship between the methylated and unmethylated groups, *q* test was used to compare the relationship between the two genes. A level of *P <* 0.05 was considered significant.

**Table 1:** Associations between SULF2, WRN methylation and clinicopathological factors in gastric tumor

**Clinicopathologic characteristics**	**n**	**WRN methylated**	**WRN unmethylated**	***P *** **value**	**SULF2 methylated**	**SULF2 unmethylated**	***P *** **value**
Age(Years old)							
> = 60	61	15	46	0.758	15	46	0.294
<60	41	9	32		14	27	
sex							
Male	75	17	58	0.732	22	53	0.736
Female	27	7	20		7	20	
Histology type							
Adenocarcinoma	81	20	61	0.659	25	56	0.336
Mucin cells or Signet ring cell type	20	4	16		4	16	
Grade							
Grade I	4	1	3	0.954	2	2	0.875
Grade II	54	16	38		20	34	
Grade III	44	7	37		7	37	
Lymphatic invasion							
Without lymphatic invasion	27	5	22	0.474	6	21	0.404
With lymphatic invasion	75	19	56		23	52	
TNM stage							
I-II	39	7	32	0.296	9	30	0.346
III-IV	63	17	46		20	43	

A level of p < 0.05 was considered significant.

## Results

### The correlation between SULF2, WRN Methylation and chemosensitivity of irinotecan in gastric cancer

**Figure 1:**
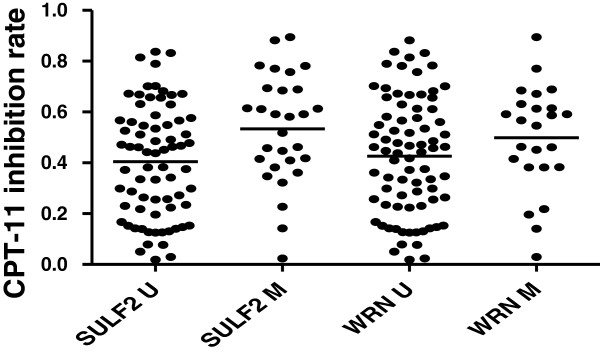
**The relationship between SULF2, WRN promoter methylation and the sensitivity for gastric cancer to Irinotecan SULF2 U: SULF2 unmethylation SULF2 M: SULF2 methylation.** WRN U: WRN unmethylation WRN M: WRN methylation.

**Figure 2:**
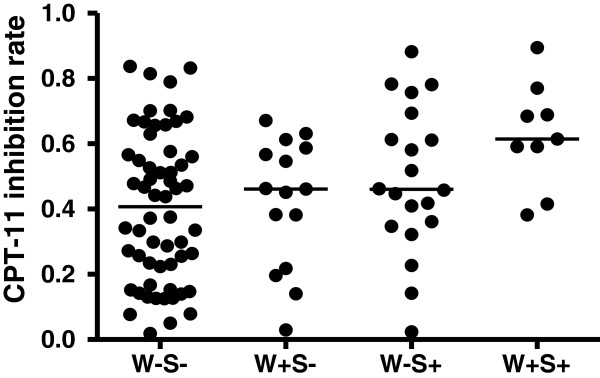
**The relationship between combined WRN, SULF2 promoter methylation and the sensitivity for gastric cancer to irinotecan.** W-S-: WRN unmethylation、SULF2 unmethylation; W + S-: WRN methylation、SULF2 unmethylation. W-S + : WRN unmethylation、SULF2 methylation; W + S+: WRN methylation、SULF2 methylation.

The inhibition rates were 0.7061 ± 0.0872 for the sensitive group (30 samples, 30.0%) and 0.3337 ± 0.1647 for the resistant group (72 samples,70%); 0.5335 ± 0.2140 for the SULF2 methylation,and 0.4045 ± 0.2197 for the SULF2 unmethylation (*χ*^2^ = 6.945, *P* = 0.008). And 0.4988 ± 0.2061 for the WRN methylation, 0.4261 ± 0.2282 for the WRN unmethylation (*χ*^2^ = 1.545, *P* = 0.214) (Figure 1). And we compared the relationship between the two genes. The inhibition rates were 0.4034 ± 0.2266 for the 58 W-S- cases, 0.6258 ± 0.1605 for the 9 S + W + cases, 0.4920 ± 0.2254 for the 20 S + W- cases, 0.4227 ± 0.1961 for the 15 S-W + cases, (*P* = 0.0287) (Figure 2). This result indicated that gastric cancer patients were more sensitive to CPT-11 when SULF2 was methylated.

## Discussion

In advanced gastric cancer, chemotherapy is the standard treatment over the world, because it prolongs patients’ survival compared to best supportive care alone. Nevertheless, the outcome of advanced stage gastric cancer still remains poor with currently available treatments, and a worldwide consensus on the standard chemotherapy has not been established. The most widely used chemotherapeutics drugs are 5-fluorouracil, adriamycin, docetaxel, cisplatin, irinotecan single-agent or combination [6]. Irinotecan, an inhibitor of topoisomerase I, acting through the inhibition of DNA replication, has emerged importantly in the treatment of patients with digestive tumors, specifically in colorectal tumors. Recent years, irinotecan has demonstrated its potential efficacy in gastric cancer with 14–23% RR as single agent [2]. Future challenges lie with how to optimize personalized therapies by incorporating molecular biomarkers in clinical practice to guide targeted therapeutics in adjunct to current standards of care.

CpG island methylation is an important epigenetic mechanism through silencing of tumor genes [7]. Nowadays, many tumor genes have been demonstrated to be inactivated by promoter hypermethylation in gastric carcinoma [8].

SULF2 is oncogene,it promotes the release of growth and angiogenic factors such as FGF-I, FGF −2, VEGF and DSF –I by mobilizing heparin-bound growth factors, leading to increased tumor growth and a more rapid rate of tumor recurrence after surgery [9,10]. In some breast, lung, liver cancers it is over expressed [11,12]. Desai SD found,silencing SULF2 through small interfering RNA or methylation primarily increased expression of interferon-inducible genes including ISG15, a marker for increased sensitivity to topoisomerase-1 inhibitors such as camptothecin (CPT) [13]. Previous studies have demonstrated that NSCLC cell lines with methylated SULF2 ( SULF2M) express 60-fold higher ISG15 compared with SULF2 unmethylated (SULF2U ) NSCLC cell lines and normal human bronchial epithelial cells. In vitro, SULF2M and high ISG15 expressing NSCLC cell lines were 134-fold more sensitive to CPT than SULF2U and low ISG15 expressing cell lines [3]. Tessema used 175 primary lung adenocarcinomas, and survival data was available for these patients. Consistent with the observation, the median survival of patients without or with SULF2 methylation was 35.1 and 62.8 months, respectively. The most dramatic effect of SULF2 methylation on survival was observed in advanced stage patients, the median survival was increased to 4-fold from 8.5 months in patients with unmethylated SULF2 to 36.2 months in patients with methylated SULF2, *P* = 0.001. In a word, it is identified a prognostic biomarker for better survival of lung cancer patients [14].

WRN has been proposed as a tumor-suppressor gene. It’s required for cellular DNA replication, mismatch repair, double strand breaks repair, and has an exonuclease,recombination activity [15]. It is localized at 8p11.2–p12 in many tumor types, including breast and colorectal cancer [16,17]. WRN hypermethylation in epithelial tumors was most prevalent in colorectal cancer (37.9%, 69/182), followed by non-small cell lung (37.5%, 21 /56), gastric (25%, 10/38), prostate (20%, 4/20), breast (17.2%, 10/58), and thyroid (12.5%, 4/32) tumors [4]. Agrelo selected a number of WRN hypermethylated (n = 45) and unmethylated (n = 43) primary colorectal tumors from patients treated with irinotecan, from whom an exhaustive clinical survival data was collected. They found that the overall survival of patients was 39.4 months for patients with WRN methylated but only 20.7 months for WRN unmethylated colon tumors. These findings underline the significance that the presence of WRN CpG island promoter hypermethylation was an important predictor of increased overall survival in colon cancer patients treated with irinotecan (*P* = 0.00005) [4].

Based on previous studies,to analyze the methylation status of the promoter-associated CpG island of both WRN and SULF2, we collected 102 samples of primary gastric tumors. The chemosensitivity testing method we adopted here is HDRA, which is a useful predictor of response to chemotherapy at different cancerous sites, mainly gastrointestinal cancer [18,19]. Taken together, our study is the first to demonstrate that SULF2 methylation was associated with a higher chemosensitivity to CPT-11,but WRN was not related. Maybe different tumor cell types have different biological behaviors, and lead to different experimental results.. What makes the case even more interesting is that gastric cancer appearing with both SULF2 and WRN methylation is remarkably more sensitive with CPT-11. It may provide further insight into guide selection of effective chemotherapeutic agents.

In our study, we did not find a statistically significant association between their promoter methylation and clinicopathological parmeters of gastric cancer. Larger prospective studies would be necessary to further validate these findings. Moreover, the experiment on tissue sample might not be representative of the biological behavior of the patient’s tumor. Therefore,it is worth our observations including tumor response, survival overall, clinical symptoms, or occurrence of treatment-associated adverse events. Also, we need to evaluate the expression of ISG15 of the tumors with SULF2 overexpressed about the sensitivity to TOPO1 inhibitors. Future studies including more genes (such as TOP-I [20], PARP [21], and Aprataxin(APTX) [22]) will be carried out to identify and target the most sensitive gastric cancer subpopulation for personalized CPT-11 therapy.

## Conclusions

We collected 102 pathologically diagnosed gastric tumor tissues. Our experiment demonstrated that SULF2 CpG island methylation renders gastric tumors sensitive to irinotecan. Besides, tumors appearing with both SULF2 and WRN methylation are more sensitive to CPT-11. It may help us to identify and target the most sensitive gastric cancer subpopulation for personalized CPT-11 therapy.

## Competing interests

The authors declare that they have no competing interests.

## Authors’ contributions

LW carried out the experimental work and collection of data, analysis and interpretation of results, drifting and substantial editing the manuscript. LX carried out the conception and participation in design. JW participated in the conception and participation in design, analysis and interpretation of results. JS participated in the collection of data, analysis and interpretation of results. BL participated actively in the analyses of the plan and provided critical reviews on the manuscripts. All authors read and approved the final manuscript.

## Pre-publication history

The pre-publication history for this paper can be accessed here:

http://www.biomedcentral.com/1471-230X/13/173/prepub

## References

[B1] Jemal A Bray F Center MM Global cancer statistics CA Cancer J Clin 2011 13 69 90 10.3322/caac.20107 21296855

[B2] Fadi SF A general review of the role of irinotecan (CPT11) in the treatment of gastric cancer Med Oncol 2007 13 137 146 10.1007/BF02698032 17848736

[B3] Tessema M Yingling CM Thomaa CL SULF2 methylation is prognostic for lung cancer survival and increases sensitivity to topoisomerase-I inhibitors via induction of ISG15 Oncogene 2011 13 1 10 10.1038/onc.2011.577 PMC3307938 22158045

[B4] Agrelo R Cheng WH Fernando S Epigenetic inactivation of the premature aging Werner syndrome gene in human cancer Proc Natl Acad Sci U S A 2006 13 8823 8827 10.1073/pnas.0600645103 PMC1466544 16723399

[B5] Yoshimasu T Oura S Hirai I Data acquisition for the histoculture drug response assay in lung cancer J Thorac Cardiovasc Surg 2007 13 303 308 10.1016/j.jtcvs.2006.06.030 17258552

[B6] TETSURO K Chemotherapy strategies for gastric cancer In Vivo 2008 13 273 278 18610735

[B7] Das PM Singal R DNA methylation and cancer J Clin Oncol 2004 13 4632 4642 10.1200/JCO.2004.07.151 15542813

[B8] Rashid A Issa JP CpG island methylation in gastroenterologic neoplasia: a maturing field Gastroenterology 2004 13 1578 1588 10.1053/j.gastro.2004.09.007 15521024

[B9] Uchimura K Morimoto-Tomita M Bistrup A Sulf-2, an extracellular endoglucosamine-6-sulfatase, selectively mobilizes heparin-bound growth factors and chemokines: effects on VEGF, FGF-1, and SDF-1 BMC Biochem 2006 13 2 14 10.1186/1471-2091-7-2 16417632 PMC1386684

[B10] Lai JP Sandhu DS Yu C Han T Moser CD Jackson KK Sulfatase 2 up-regulates glypican 3, promotes fibroblast growth factor signaling, and decreases survival in hepatocellular carcinoma Hepatology 2008 13 1211 1222 10.1002/hep.22202 18318435 PMC2536494

[B11] Morimoto-Tomita M Uchimura K Bistrup A Lum DH Egeblad M Boudreau N Sulf-2, a proangiogenic heparin sulfate endosulfatase, is upregulated in breast cancer Neoplasia 2005 13 1001 1010 10.1593/neo.05496 16331886 PMC1502017

[B12] Lemjabbar-Alaoui H van Zante A Singer MS Xue Q Wang YQ Tsay D Sulf-2, a heparan sulfate endosulfatase, promotes human lung carcinogenesis Oncogene 2010 13 635 646 10.1038/onc.2009.365 19855436 PMC2818095

[B13] Desai SD Wood LM Tsai YC ISG15 as a novel tumor biomarker for drug sensitivity Mol Cancer Ther 2008 13 1430 1439 10.1158/1535-7163.MCT-07-2345 18566215 PMC2561335

[B14] Tessema M Yu YY Stidley CA Machida EO Schuebel KE Baylin SB Concomitant promoter methylation of multiple genes in lung adenocarcinomas from current, former and never smokers Carcinogenesis 2009 13 1132 1138 10.1093/carcin/bgp114 19435948 PMC2704285

[B15] Agrelo R Cheng WH Setien F Epigenetic inactivation of the premature aging Werner syn-drome gene in human cancer Proc Natl Acad Sci U S A 2006 13 8822 8827 10.1073/pnas.0600645103 16723399 PMC1466544

[B16] Wen-Hsing C Rika K Patricia L Opresko Collaboration of Werner syndrome protein and BRCA1 in cellular responses to DNA interstrand cross-links Nucleic Acids Res 2006 13 92751 92760 10.1093/nar/gkl362 PMC1464112 16714450

[B17] Monika A Joshua A Sommers Robert H Hoemaker, inhibition of helicase activity by a small molecule impairs Werner syndrome helicase (WRN) function in the cellular response to DNA damage or replication stress Proc Natl Acad Sci U S A 2011 13 40 1525 1530 21220316 10.1073/pnas.1006423108 PMC3029756

[B18] Furukawa T Kubota T Hoffman RM Clinical applications of the histoculture drug response assay Clin Cancer Res 1995 13 305 311 9815986

[B19] Hayashi Y Kuriyama H Umezu H Tanaka J Yoshimasu T Furukawa T Class III beta-tubulin expression in tumor cells is correlated with resistance to docetaxel in patients with completely resected non-small-cell lung cancer InternMed 2009 13 203 208 10.2169/internalmedicine.48.1659 19218769

[B20] Koopman M Knijn N Richman S The correlation between topoisomerase-I (Topo1) expression and outcome of treatment with capecitabine and irinotecan in advanced colorectal cancer (ACC) patients (pts) treated in the CAIRO study of the Dutch Colorectal Cancer Group (DCCG) Eur J Cancer 2009 13 321 325

[B21] Hoskins JM Marcuello E Altes A Irinotecan pharmacogenetics: influence of pharmacodynamic genes Clin Cancer Re 2008 13 1788 1796 10.1158/1078-0432.CCR-07-1472 18347181

[B22] Higinio D Silvia ML Elena E Aprataxin tumor levels predict response of colorectal cancer patients to irinotecan-based treatment Clin Cancer Res 2010 13 8 2375 2382 10.1158/1078-0432.CCR-09-3275 20371676

